# Urinary antigen–negative Legionella pneumonia in critically ill patients: value of systematic molecular testing for optimal antimicrobial therapy

**DOI:** 10.1093/jac/dkag101

**Published:** 2026-03-14

**Authors:** Livia Foglia Manzillo, Maddalena Teggia Droghi, Francesca Iannuzzi, Daniele Castelli, Giuseppe Foti, Paolo Bonfanti, Marco Giani

**Affiliations:** Department of Medicine and Surgery, University of Milano-Bicocca, Monza, Italy; Emergency and Intensive Care, Fondazione IRCCS San Gerardo, Monza, Italy; Emergency and Intensive Care, Fondazione IRCCS San Gerardo, Monza, Italy; Infectious Diseases, Fondazione IRCCS San Gerardo, Monza, Italy; Microbiology, Fondazione IRCCS San Gerardo, Monza, Italy; Department of Medicine and Surgery, University of Milano-Bicocca, Monza, Italy; Emergency and Intensive Care, Fondazione IRCCS San Gerardo, Monza, Italy; Department of Medicine and Surgery, University of Milano-Bicocca, Monza, Italy; Infectious Diseases, Fondazione IRCCS San Gerardo, Monza, Italy; Department of Medicine and Surgery, University of Milano-Bicocca, Monza, Italy; Emergency and Intensive Care, Fondazione IRCCS San Gerardo, Monza, Italy

Dear Editor,

Legionnaires’ disease, often underdiagnosed, requires intensive care unit (ICU) admission in nearly one-third of cases with substantial mortality.^1^ Culture isolation, although the diagnostic standard, is impractical in critically ill patients because of slow turnaround and suboptimal sensitivity. Serology has limited utility for similar reasons.^2^ Consequently, the initial diagnostic approach largely relies on the rapid and inexpensive urinary antigen test (UAT), which detects only L. pneumophila serogroup 1 (SG1) and therefore fails to identify other serogroups or species.^3^ Limited sensitivity may reflect assay performance, non-SG1 strains, or previous antimicrobial therapy, and may lead to unnecessary prolonged broad-spectrum therapy. Faster and more sensitive molecular diagnostic tools—particularly polymerase chain reaction (PCR) assays—have been increasingly implemented for Legionella diagnosis.^2^

At our institution, systematic molecular testing of all respiratory specimens by PCR was introduced in January 2024 for patients with respiratory failure. Since then, we have observed several cases of Legionella pneumonia with negative UAT but positive PCR results on bronchoalveolar lavage (BAL) samples.

To investigate this finding, we retrospectively analysed all severe Legionella pneumonia cases admitted to our ICU, focusing on the prevalence and implications of UAT-negative infections.

Between January 2024 and October 2025, we identified all ICU admissions for severe Legionella pneumonia, defined as acute respiratory failure requiring invasive mechanical ventilation or advanced organ support. Demographic and clinical variables, severity scores, laboratory results, ventilatory parameters, antibiotic therapy, and ICU outcomes were collected. On admission, patients underwent fibreoptic bronchoscopy for BAL samples. BAL specimens were analysed with multiplex PCR (Biofire^®^ FilmArray^®^, Biomerieux, Marcy-l'Étoile, France) testing for rapid pathogen identification, with parallel cultures performed to provide confirmatory or complementary microbiological data.

Sixteen patients with severe Legionella pneumonia were included during the study period. The cohort consisted of 11 men and 5 women, with a mean age of 67 years. The median SOFA score on ICU admission was 8 (6.25–11.5), and the median PaO_2_/FiO_2_ ratio was 115 (92–134.75). All patients required invasive mechanical ventilation, and three received venovenous extracorporeal membrane oxygenation.

BAL samples tested positive for Legionella spp. by PCR within 3–4 hours. Five patients (31%) had a negative UAT on admission; all were male, with no epidemiological links identified. Table 1 summarizes baseline clinical, laboratory, and ventilatory characteristics according to UAT status, which did not differ significantly among groups. Serum sodium was lower in UAT-positive patients (*P* = 0.052). All patients received empiric broad-spectrum antibiotic therapy, typically a β-lactam combined with a macrolide or fluoroquinolone, with non-β-lactam-target coverage maintained until PCR confirmation. Following PCR confirmation, therapy was refined according to our institutional approach for the most severe cases: fifteen patients received the combination therapy of azithromycin and levofloxacin,^4^ and one patient received azithromycin alone. This allowed early discontinuation of empiric agents, consistent with antimicrobial-stewardship principles. The appropriate antibiotic regimen was maintained for a total course of 14–21 days. All patients were successfully weaned from mechanical ventilation and survived to ICU discharge.

**Table 1.** Baseline clinical, laboratory, and ventilatory characteristics of patients according to urinary antigen test status

Variable	Antigen-Positive (*n* = 11)	Antigen-Negative (*n* = 5)	*P* value
Age, years	64 (54–74)	64 (57–77)	0.777
PaO_2_/FiO_2_ ratio	116 (77–149)	114 (92–134)	0.777
SOFA score	7 (5–12)	8 (7–10)	0.648
WBC count, ×10^9^/L	9.5 (4.4–25.2)	13.7 (8–17.7)	0.571
CRP, mg/L	47 (32–281)	100 (37–296)	0.865
PCT, ng/mL	12 (3–75)	5.5 (1.8–12.8)	0.171
Creatinine, mg/dL	0.8 (0.65–1.95)	0.95 (0.65–1.40)	0.864
Sodium, mmol/L	136 (134–140)	140 (138–146)	0.052
CPK, U/L	270 (50–9805)	95 (27–161)	0.192
PEEP, cmH_2_O	10 (6–13)	11 (9–14)	0.227
Crs, mL/cmH_2_O	46 (44–66)	45 (25–66)	0.691
Ventilatory Ratio	0.9 (0.35–1.04)	1.3 (0.85–1.70)	0.152

Values are expressed as median (interquartile range). *P* values refer to comparisons between Ag-positive (*n* = 11) and Ag-negative (*n* = 5) patients.

CPK, creatine phosphokinase; CRP, C-reactive protein; Crs, respiratory system compliance; PaO_2_/FiO_2_, arterial oxygen tension/inspired oxygen fraction ratio; PCT, procalcitonin; PEEP, positive end-expiratory pressure; SOFA, Sequential Organ Failure Assessment; WBC, white blood cell count.

In this series of critically ill patients with Legionella pneumonia requiring invasive mechanical ventilation, systematic molecular testing identified nearly one-third of cases as UAT-negative, enabling timely diagnosis and targeted therapy. Legionella pneumonia remains associated with considerable ICU mortality.^1^ Thus, timely diagnosis and appropriate antibiotic therapy are crucial. Although UAT is widely adopted for its simplicity, it relies on low-sensitivity assays and only detects L. pneumophila SG1, thus missing other serogroups or species. In Italy, most reported cases rely solely on UAT, with limited use of culture or molecular methods. PCR-based assays have emerged as rapid and highly sensitive diagnostic tools, though they may identify non-viable organisms, contributing to imperfect concordance with culture.^5^ Furthermore, in our workflow, PCR assays that were implemented only detect L. pneumophila, albeit not limited to SG1.

Our findings highlight the value of systematic molecular testing within a rapid molecular diagnostic workflow for critically ill patients with severe pneumonia. Routine PCR on BAL samples enabled prompt identification of Legionella spp. in UAT-negative cases, ensuring timely initiation and continuation of targeted therapy. Without a multiplex syndromic PCR panel, empiric macrolide or fluoroquinolone therapy might have been discontinued prematurely, while broad-spectrum agents—often including carbapenems—would likely have been prolonged. Early microbiological confirmation supported antimicrobial stewardship by facilitating de-escalation and reducing avoidable toxicity and selective pressure for resistance.

This study is limited by its single-centre, retrospective design, small sample size, which may restrict generalizability, and lack of systematic serogroup typing. While prior therapy before ICU admission may reduce UAT sensitivity, the repeated identification of UAT-negative Legionella infections supports the clinical value of systematic molecular testing. When bronchoscopy is not feasible, reliance on UAT alone may lead to missed diagnoses and inappropriate antibiotic management. Adoption of broader diagnostic strategies that incorporate molecular assays alongside conventional methods may improve diagnostic accuracy, antimicrobial stewardship, and outcomes in severe community-acquired pneumonia.

## Data Availability

The datasets generated and/or analysed during the current study are available from the corresponding author (M.G.) upon reasonable request.

## References

[1] Rello J, Allam C, Ruiz-Spinelli A et al Severe Legionnaires’ disease. Ann Intensive Care 2024; 14: 51. 10.1186/s13613-024-01252-y 38565811 PMC10987467

[2] Ricci ML, Grottola A, Fregni Serpini G et al Improvement of Legionnaires’ disease diagnosis using real-time PCR assay: a retrospective analysis, Italy, 2010 to 2015. Euro Surveill 2018; 23: 1800032. 10.2807/1560-7917.ES.2018.23.50.1800032 30563592 PMC6299505

[3] Metlay JP, Waterer GW, Long AC et al Diagnosis and treatment of adults with community-acquired pneumonia. An official clinical practice guideline of the American Thoracic Society and Infectious Diseases Society of America. Am J Respir Crit Care Med 2019; 200: e45–67. 10.1164/rccm.201908-1581ST 31573350 PMC6812437

[4] Martin SJ, Pendland SL, Chen C et al In vitro synergy testing of macrolide-quinolone combinations against 41 clinical isolates of Legionella. Antimicrob Agents Chemother 1996; 40: 1419–21. 10.1128/AAC.40.6.1419 8726012 PMC163342

[5] Falcone M, Russo A, Tiseo G et al Predictors of intensive care unit admission in patients with Legionella pneumonia: role of the time to appropriate antibiotic therapy. Infection 2021; 49: 321–5. 10.1007/s15010-020-01565-7 33315182 PMC7734452

